# Supplementary figures and images for: HTL/KAI2 signaling substitutes for light to control plant germination
Source: PLoS Genet. 2024 Oct 21;20(10):e1011447. doi: 10.1371/journal.pgen.1011447 (PMC11527322; doi:10.1371/journal.pgen.1011447)

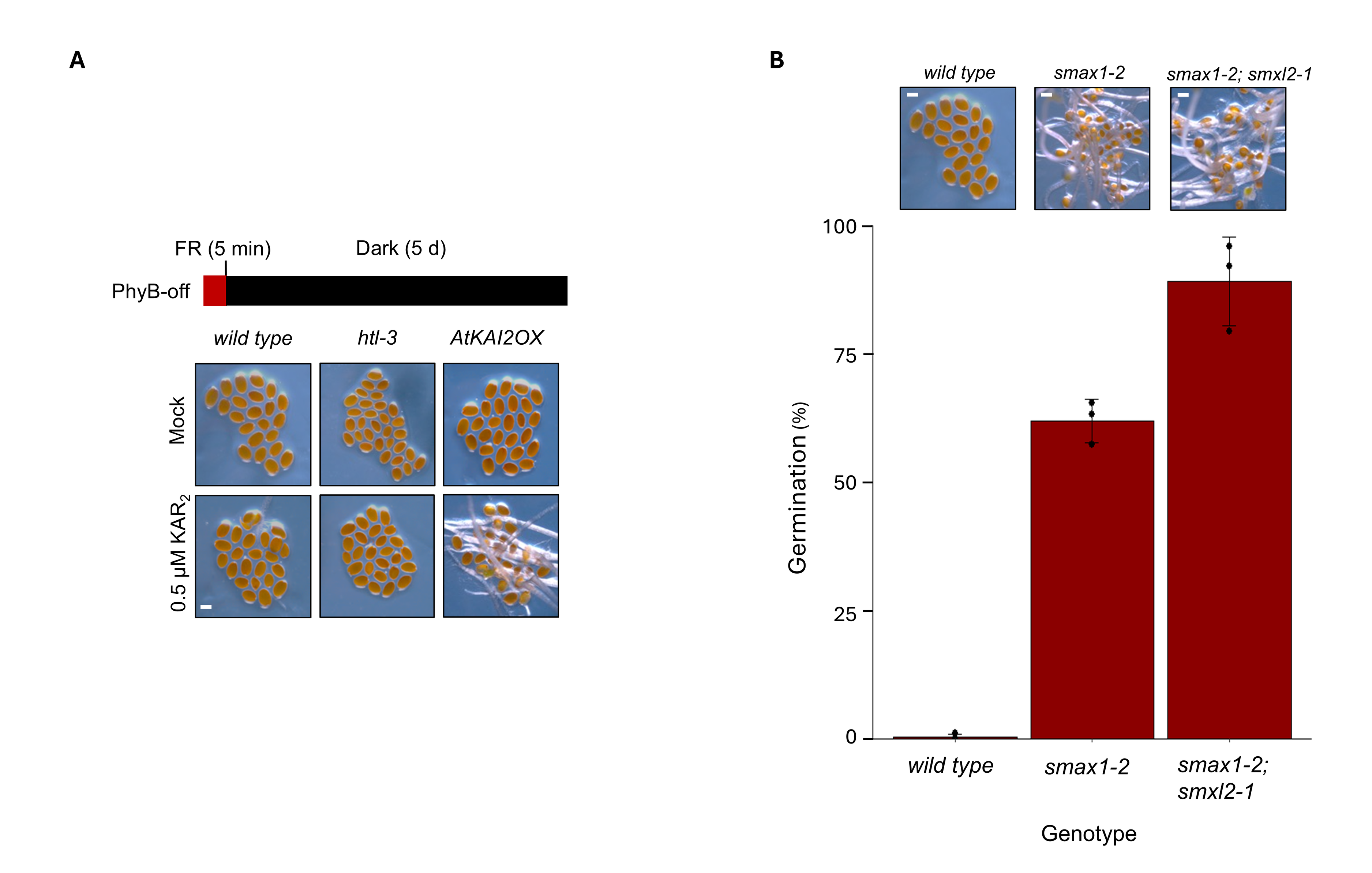

Supplement: S1 Fig — (A) Representative images of seeds treated with or without 0.5 μM KAR2 after five minutes of far-red light exposure and placed in a room-temperature dark cabinet. (B) Germination percentages of seeds after a far-red light regime as shown in (A). Germination was counted as radicle emergence. Bar plot represents three biological replicates. Black circles represent the mean of each biological replicate. Bars represent SD. (TIF) [file pgen.1011447.s001.tif]

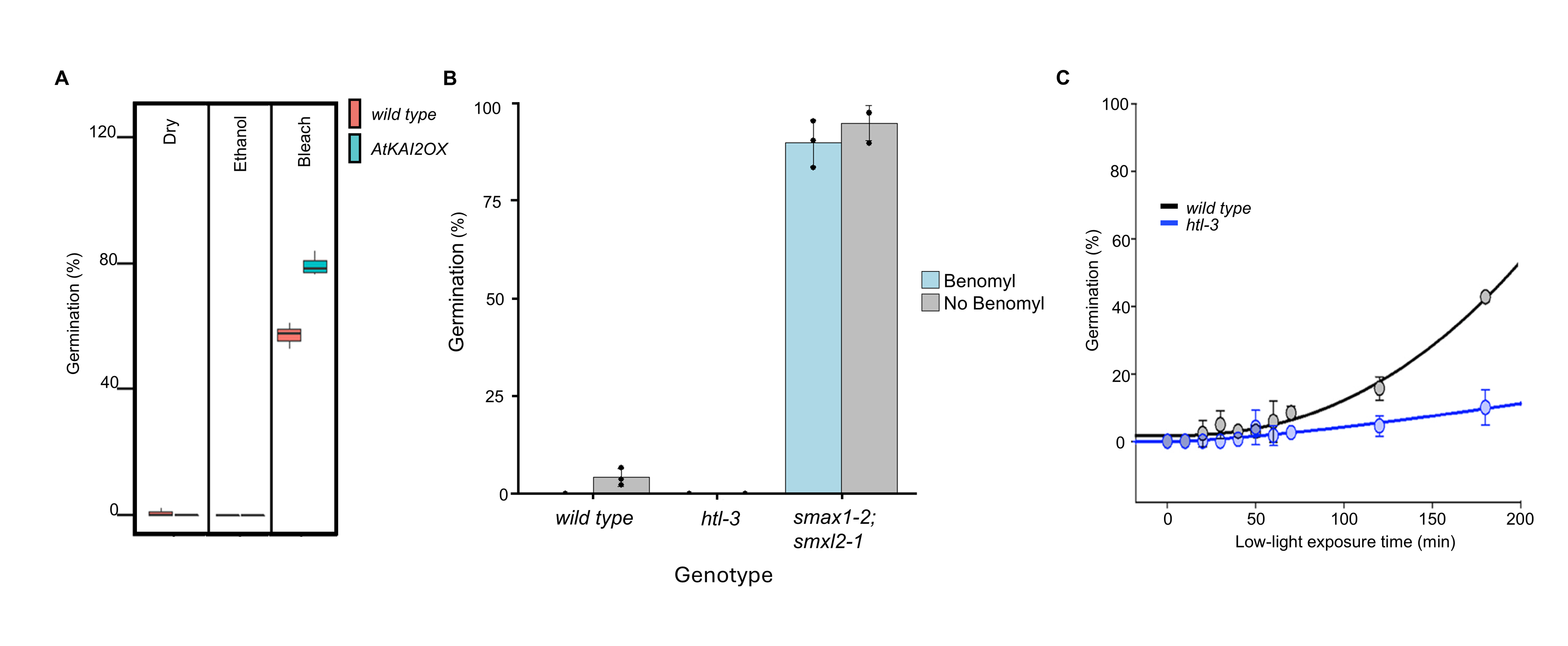

Supplement: S2 Fig — (A) Germination of Arabidopsis seed under different sterilization conditions in the dark. (B) Germination percentages in the dark with or without the fungicide, benomyl. Germination was counted as radicle emergence. Bar plot represents three technical replicates. Closed circles represent the mean of each biological replicate. Bars represent SD. (C) Germination under different lengths of exposure to low light (0.3 μmol m-2 s-1). Sample sizes and box plot elements are provided in S1 Table. (TIF) [file pgen.1011447.s002.tif]

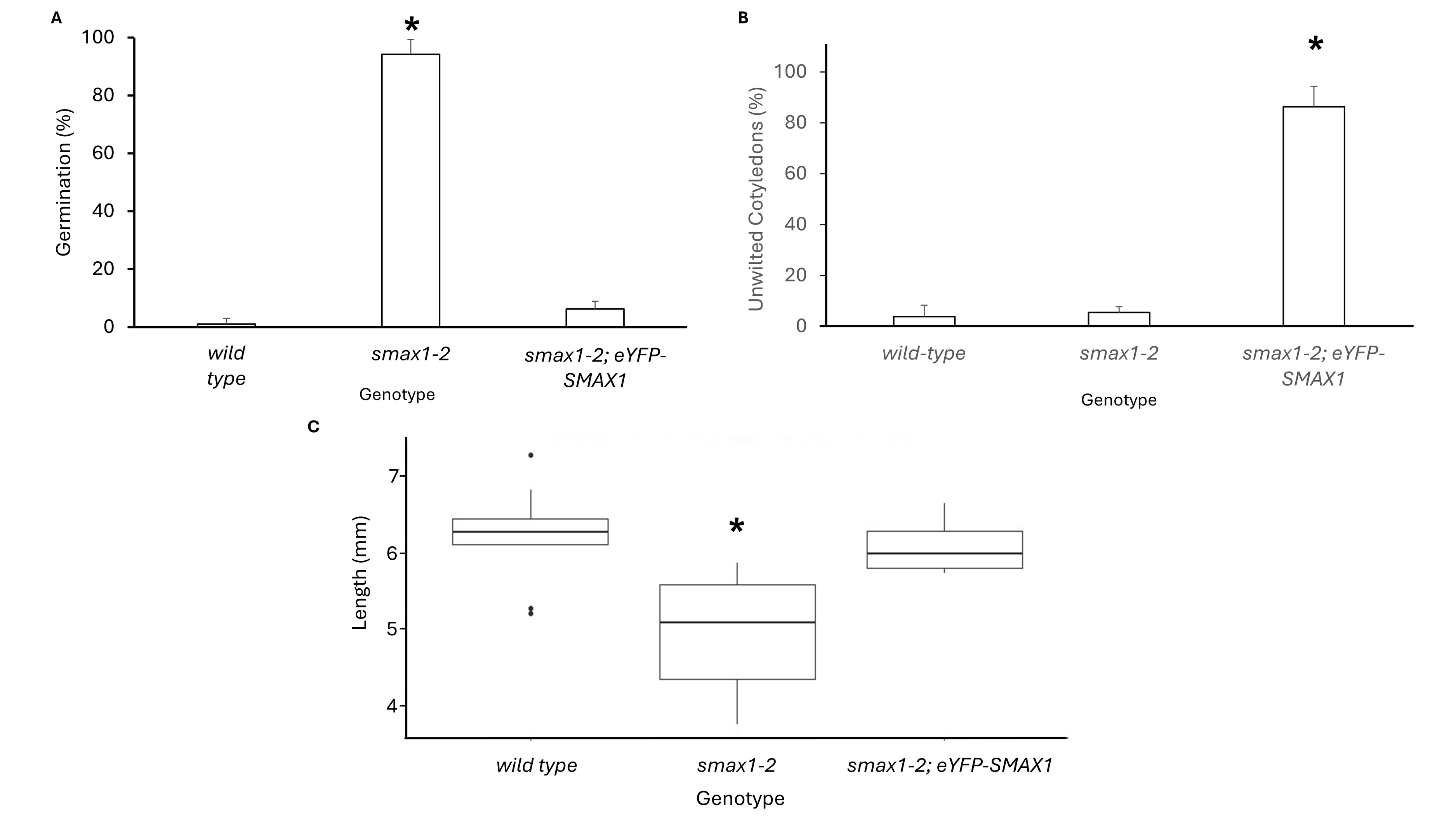

Supplement: S3 Fig — (A) 35S::eYFP-SMAX1 functionally complements smax1-2 on 20 μM Paclobutrazol (PAC). Germination on 20 μM PAC of wild type, smax1-2 and smax1-2; 35S::eYFP-SMAX1 transgenic line. Average germination was counted for radicle emergence of three technical replicates. Bar = SD. Asterisks indicate a significant difference to wild type (p<0.05, ANOVA one-way with post-hoc Tukey HSD test). (B) Overexpression of eYFP-SMAX1 is resistant on 20 μM hygromycin. Germination on hygromycin of wild type, smax1-2 and smax1-2; 35S::eYFP-SMAX1 transgenic line. Resistance was counted as unwilted cotyledons of three technical replicates. Bars represent SD. Asterisks indicate a significant difference to wild type (p<0.05, ANOVA one-way with post-hoc Tukey HSD test. (C) Overexpression of eYFP-SMAX1 functionally complements smax1-2 hypocotyl length. Hypocotyl length of wild-type, smax1-2, and smax1-2; 35S::eYFP-SMAX1 transgenic line. Seedlings were stratified for four days and then grown under white light for seven days. Hypocotyl length was measured with ImageJ. N = 10–11. Representative seedlings displayed above boxplot in order of x-axis. Bar = 1mm. Asterisks indicate a significant difference to wild type (p<0.05, ANOVA one-way with post-hoc Tukey HSD test). P values, sample sizes and box plot elements are provided in S1 Table. (TIF) [file pgen.1011447.s003.tif]

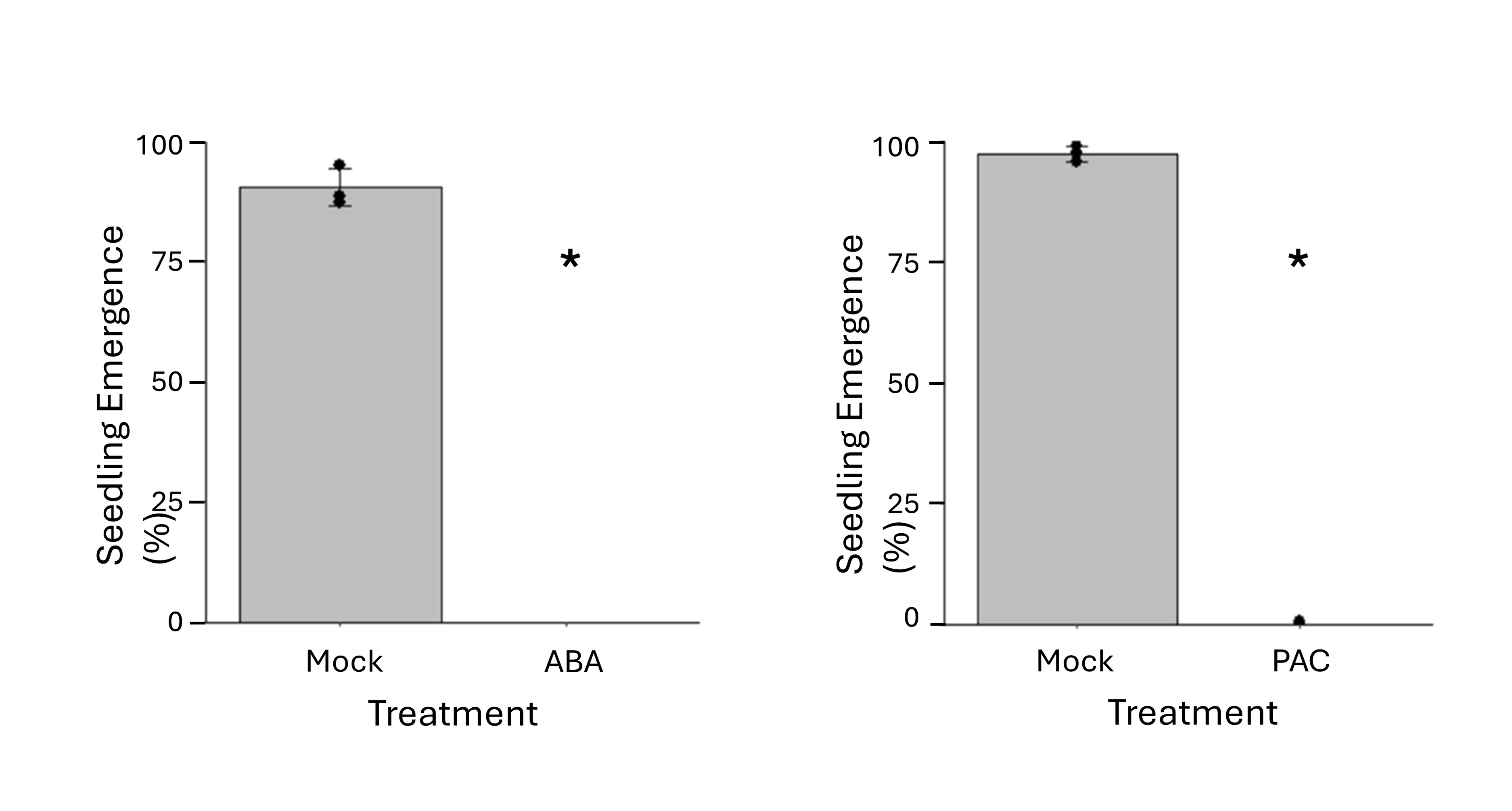

Supplement: S4 Fig — Seedling emergence was quantified as fully elongated hypocotyls. Black circles represent the mean of each biological replicate. Bar = SD. Asterisks indicate a significant difference compared to untreated (mock) smax1-2; smxl2-1 seeds (P < 0.05, one-way ANOVA with post-hoc Tukey honest significant difference test). P values, sample sizes and box plot elements are provided in S1 Table. (TIF) [file pgen.1011447.s004.tif]

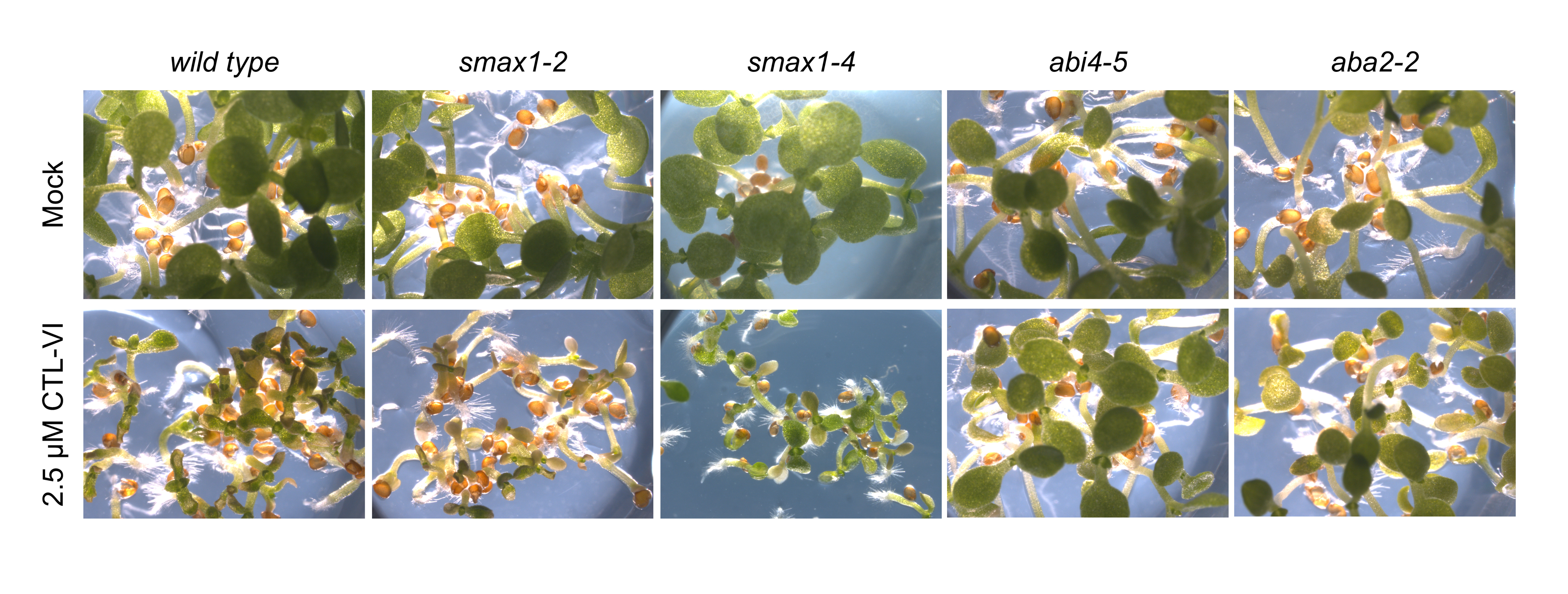

Supplement: S5 Fig — Representative images of dark germination mutants germination with or without 2.5 μM CTL-VI. (TIF) [file pgen.1011447.s005.tif]

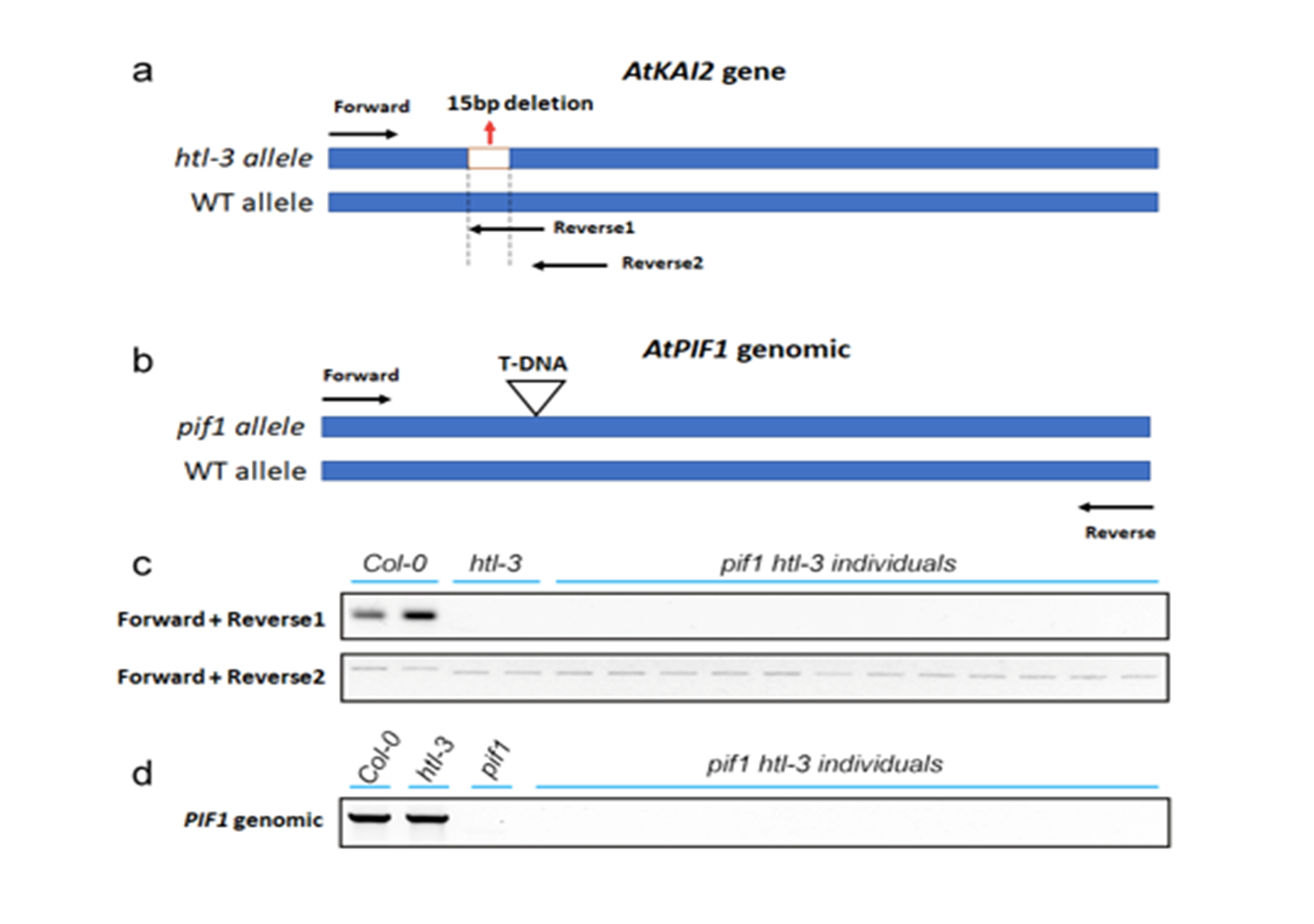

Supplement: S6 Fig — (A) Schematic illustration of WT and htl-3 allele of AtKAI2 gene, as well as the primers for htl-3 genotyping. (B) Schematic illustration of WT and pif1 T-DNA insertion allele of AtPIF1 gene, as well as the primers for pif1 allele genotyping. (C) Confirmation of htl-3 allele in individual pif1 htl-3 double mutants. (D) Confirmation of pif1 allele in individual pif1; htl-3 double mutants. (TIF) [file pgen.1011447.s006.tif]
