# Supplementary material for: HTL/KAI2 signaling substitutes for light to control plant germination
Source: PLoS Genet. 2024 Oct 21;20(10):e1011447. doi: 10.1371/journal.pgen.1011447 (PMC11527322; doi:10.1371/journal.pgen.1011447)
Supplement: Attachment — Submitted filename: sl_PlosGenetics_Response_Reviewers_V1.pdf [file pgen.1011447.s016.pdf]

Reviewer #1:

In the revised manuscript, the authors have addressed my concerns to my satisfaction. I appreciate the new Figure 6 and the mechanistic explanation for the differential effects of PAC in light vs dark. I think the comments raised by the other reviewers have also been well addressed, and the additional control experiments are welcome. There is one, however, that I think remains to be managed.

Reviewer 3 queried the logic of lines 421-425 (now lines 485-489). The first time I read this section, I also paused for thought because something seemed amiss - the analysis seemed a little too simplistic. At the time I didn't bother thinking it through properly. But seeing as reviewer #3 raised it but did not specify exactly what the problem was, causing the authors to dismiss the concern, I thought I would revisit it.

We have decided to remove this section to avoid confusion.

Reviewer #3:

Point #1 - I was asking the authors to more thoroughly cite and discuss in the manuscript the findings in the preexisting literature that are relevant to this topic (e.g. KAR signaling, light, and GA during germination). The authors' response to this point seems to be intended for a different comment.

- **We have now added multiple paragraphs on the roles of karrikin, light, and GA signaling during germination in the Introduction (Lines 81-133; References 14-40). We believe this information will supplement the model (Fig. 6) proposed in the Discussion section and give more background and perspective to the readers.**

Point #2 - Please indicate in the manuscript that not all species are positively photoblastic and provide a reference to support the claim that the majority of plants use light as a positive germination signal (while making this claim, the authors may wish to keep in mind that light signals are relevant to seeds with physiological dormancy and that several other forms of seed dormancy exist).

- **We agree with the reviewer that not all species are positively photoblastic. Thus, we included a new section summarizing nonphotoblastic, positive and negative photoblastic plants. We also discussed the connection of the KAI2 pathway in positive photoblastic plants like lettuce (Lines 81-96, 471-473). As suggested by the reviewer, we have also pointed out that *Arabidopsis* has physiological-induced dormancy and for this reason, light plays a more important role in breaking dormancy. We have provided multiple references for this section [14-23].**

Point #3 - I appreciate the experiment with far-red light being performed. It remains possible that the 5 minute far-red light treatment was of insufficient intensity and duration to turn off phytochrome (this could be tested by applying the far-red treatment, or not, after a red light treatment) but if the authors are comfortable with the experimental setup it is fine with me.

- **We agree with the reviewer that additional far-red light experiments involving dose curves of both intensity and quality of light would add to the study. We think these experiments would be appropriate for future work. As the reviewer states, they are fine if we are comfortable with the experimental setup.**

Point #21 - In the previous manuscript's lines 421-425, the authors wrote "Given that only 8.9% of the EMS mutable sites in SMAX1 produce a nonsense codon, the probability of finding two nonsense alleles (smax1-1 and smax1-4) and zero missense alleles is approximately 1/125. This suggests that our screen is relatively impervious to identifying SMAX1 missense mutations possibly due to the stringency of dark as a germination condition." As I stated before, I do not agree that this is a valid analysis or conclusion.

- **As mentioned for Reviewer 1, we have now removed this section on EMS generated *smax1* alleles.**

<u>Reviewer #4:</u>

This manuscript details the important role of SMAX1 in Arabidopsis germination in the dark and provides evidence that both SMAX1 and SMXL2 are necessary for PIF1 function under these conditions. A working model is presented to describe the contributions of SMAX1 and SMXL2 to germination in dark versus light conditions, effectively integrating prior research on KAR signaling, light, and GA during germination. These results are significant and impactful in explaining how specialized plants, such as ephemeral and parasitic weeds, have evolved their germination behavior in response to specific environments.

Major comments：
1. Fig 5, the photographs showing the bleached seedling phenotype of various mutants grown on 2.5 μM CT-VI are not clear enough, possibly due to insufficient image clarity or small size. So I don't think it is obvious that "mutants constitutive for KL signaling, like smax1-2, may show increased sensitivity to CTL-VI application compared to ABA auxotrophs with respect to cotyledon bleaching." Please present the results of CTL-VI treatment on the mutants in a separate figure.

- **We have re-exported images in Figure 5 to improve image quality, see below.**

- **We have also generated a new supplementary figure consisting of larger close-up images of the mentioned genotypes with and without CTL-VI (S5 Fig), see below.**

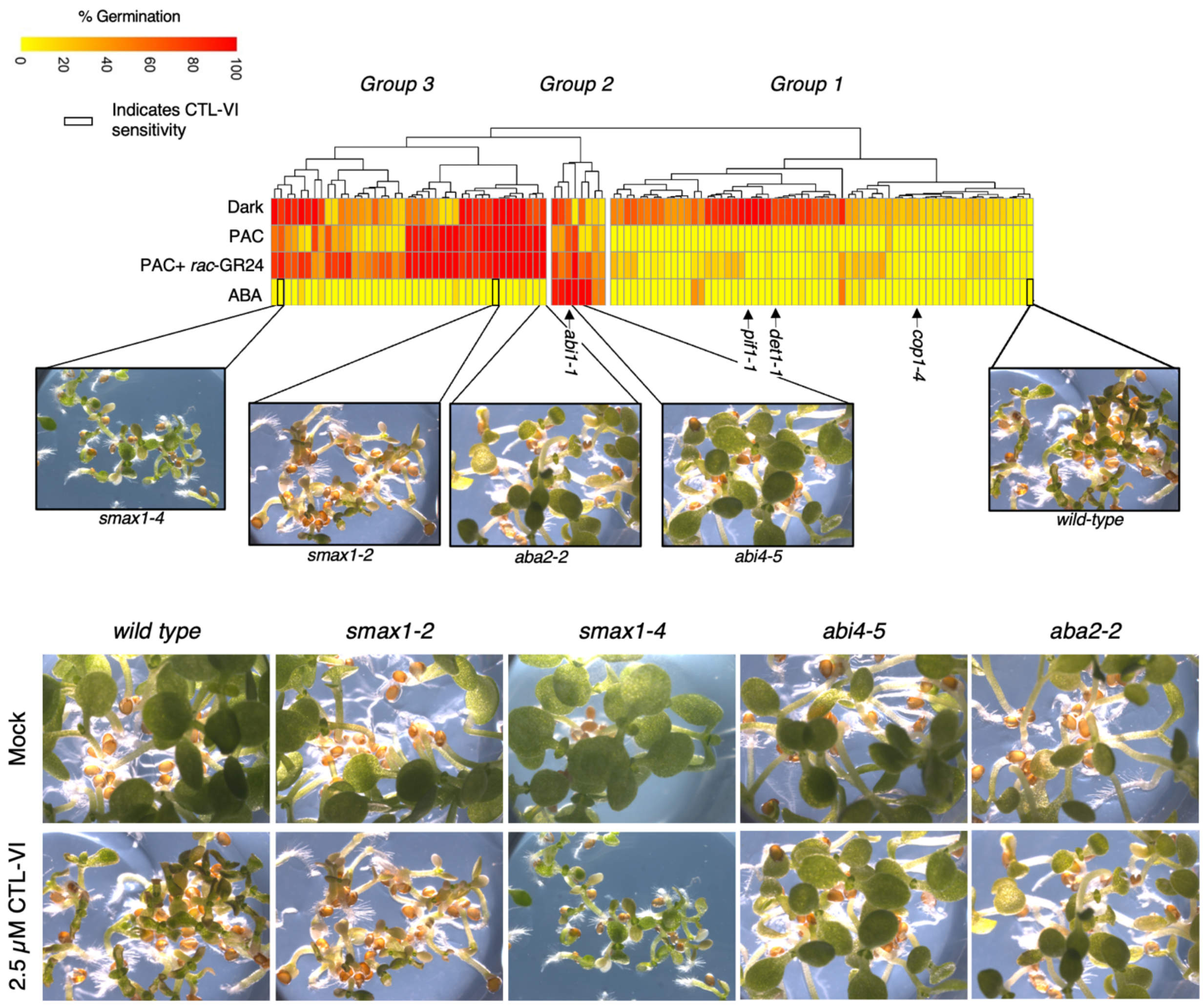

2. Why was the expression of genes known to be induced by AtKAI2 activation (DLK2, KUF1, BBX20) lower in dark-germinated AtKAI2OX-A seeds treated with DMSO than in wild-type seeds (Fig 1D)?

- **We thank the reviewer for making this interesting observation. While we do not have sufficient evidence to explain this observation, we suspect that the overexpression of *AtKAI2* could further repress expression of *DLK2*, *KUF1*, and *BBX20* compared to wild-type seeds. We would like to investigate the possibility that AtKAI2 itself has a role in repressing downstream gene expression in future work.**

3. Could the author please explain how to treat smax1-2; smxl2-1 seed with Abscisic acid (ABA) and paclobutrazol (PAC) (S4 Fig)?

- **To clarify our experimental conditions, we have added specific details to our methods under "Dark Germination Assays" (Lines 562-564), see below.**

Dark Germination Assays

Seeds for dark germination assays were left to dry for at least six months in a glass tube and closed cardboard storage box at room temperature (24°C) in the laboratory before use in germination assays. Approximately 25 to 35 of unsterilized dry seeds per replicate were sprinkled directly onto 10 mm petri plates in the dark containing water agar (0.8%) and the fungicide benomyl (10 µg/ml) (Toronto Research Chemicals, B161380). Seeds were exposed to low light (~0.1 µmol m^{-2} s^{-1}) for less than 30 seconds while plating and then plates were wrapped with three layers of aluminum foil. Plates were placed at 4˚C for four days and then incubated at 22˚C for five days in the dark. Germination was quantified using radicle emergence. Seedling emergence was quantified by counting fully elongated hypocotyls.

For all chemical treatments, rac-GR24 was used which is a racemic mixture of (+)-GR24 and (-)-GR24. A stock solution of rac-GR24, PAC (Cayman Chemical Company, 18864), and ABA (Sigma-Aldrich, A1049-100MG) was diluted in DMSO, 100% ethanol, and 100% methanol, respectively. For dark germination assays on compounds, the fungicide benomyl (10 µg/ml) and the appropriate compound at specific concentrations (3 µM ABA, 20 µM PAC) or the solvent were added to the water agar media (0.8%) at 1/1000 concentration.

4. Please explain why smax1-4 germinated well in the dark while smax1-2 did not germinate well in the dark (Fig 5).

- **We thank the reviewer for pointing out this discrepancy. We were also a bit surprised and decided to re-analyse the raw data. We discovered that Figure 5 was incorrectly labelled likely due to multiple edits of previous versions. We have gone back to our original dataset to verify the data for each row in the heat map to correct the labeling of genotypes. Our review of the data did not change the groupings, however, and *smax1-4* and *smax1-2* showed similar high germination rates in the dark as expected, see below.**

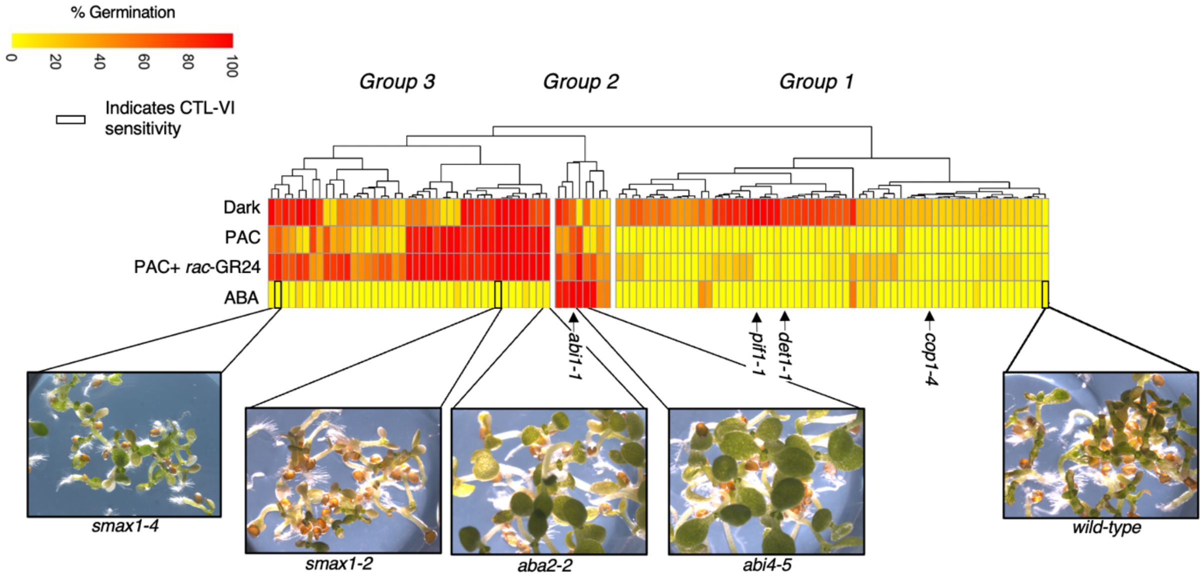

5. Line 423-424 (Fig 6) in the Revised Article with Changes Highlighted version: Which EAR repressors are recruited by SMAX1 and SMXL2 proteins? Please provide suitable references.

- **We specified which EAR repressors interact with SMAX1 and included references, "***SMAX1 and SMXL2 also could act to directly dampen germination as SMAX1 interacts with the EAR motif repressors TOPLESS1 (TPL1) and TOPLESS RELATED1, 2, 4 (TPR1, 2, 4) [59-62].*"

6. Why are smax1-2; 35S::eYFP-SMAX1 seeds sprinkled onto ½ MS (Murashige-Skoog) agar (0.8%), while the other seeds in the Dark Germination Assays are sprinkled directly onto Petri plates in the dark containing water agar (0.8%) and the fungicide benomyl (10 µg/ml)?

- ***smax1-2; 35S::eYFP-SMAX1*** **seed were imbibed on ½ MS for the purpose of imaging germinating seedlings. Under our dark germination conditions, we cannot see the root clearly and thus it was not possible to image the root.**
- **We have moved this section of text to "Confocal Microscopy" under methods.**

Minor comments：

1. Line 188 of the Revised Article with Changes Highlighted version: The reference to "S2 Fig" is incorrect. "S2 Fig" does not mention PAC treatment.

2. Line 277：S4 Fig， not S3 Fig.
3. Line 327: "Mutants perturbed in KL signaling can be identified using a compound called cotylimide-VI (CTL-VI) [36]." Should this reference be to citation 37 instead of 36? Please confirm. Ref 36 refers ABA not being able to inhibit seed coat rupture

4. Line 502：“Col-0” should not be written in italics.
5. Line 503: “smax1-2/smxl2-1 [38] pif1-1 [21]”, a comma is missing here.
6. Line 504:“abi4-5 [53], cop1-4 [53], aba2-2 [54], abi1-1 [51], det1-1 [55]”All genotypes in this sentence should be italicized.
7. Line 506: Please clarify the sentence, “All seeds were grown in a continuous 24-hour light chamber at 22°C.” What kind of photoperiod does Arabidopsis thaliana require for growth?

We have edited the sentence to, “*Arabidopsis thaliana* plants were grown in a continuous 24-hour light chamber at 22°C.” Although *Arabidopsis thaliana* is a long day plant whose flowering is delayed under short day conditions, *Arabidopsis* is commonly grown under constant light in laboratory conditions.

8. Line 509:“240 C”The unit of Celsius is misspelled.
9. Line 507-509：“Seeds for dark germination assays were left to dry for at least six months in a glass tube and closed cardboard storage box at room temperature (240 C) in the laboratory before use. “This sentence should be moved to the method part of “Dark Germination Assays”.
10. Line 535-537:“Approximately 25 to 35 of unsterilized dry seeds per replicate were sprinkled directly onto 10 mm petri plates in the dark containing water agar (0.8%) and the fungicide benomyl (10 µg/ml) (Toronto Research Chemicals, B161380). Unsterilized dry seeds were sprinkled directly onto 10 mm petri plates in the dark containing water agar (0.8%) and the fungicide benomyl (10 µg/ml) (Toronto Research Chemicals, B161380).” This passage contains a repeated sentence.

We have addressed all the above minor comments and have made all these corrections in the text.
