# Supplementary material for: HTL/KAI2 signaling substitutes for light to control plant germination
Source: PLoS Genet. 2024 Oct 21;20(10):e1011447. doi: 10.1371/journal.pgen.1011447 (PMC11527322; doi:10.1371/journal.pgen.1011447)
Supplement: Attachment — Submitted filename: sl_PLOS_Gen_Response_08.07.24_v12.docx [file pgen.1011447.s014.docx]

August 7, 2024

Dear Professors Kohler and Gutjahr,

We would like to thank you and the referees for taking the time to review our manuscript and for your patience in getting our comments back to you. We have carefully looked over the reviewers’ comments and have made changes where suggested. In summary, we **have performed germination experiments with far-red light suggested by Reviewer #3 as well as improved explanations of experiments, referencing, and integration of prior research as requested by all three reviewers.** To make it easier for the editors and referees, we have **bolded new sections**, where we substantially expanded our explanation of experiments, integrated prior research, and generated a working model.

Below, we address what we think are the two most important points raised by the reviewers before providing a point-by-point response to their other comments.

**General Comments:**

1. Reviewers wanted more explanation of how *SMAX1/SMXL2* signaling permits GA-independent germination in the light but requires GA in the dark. They also would like an integration of our study with the roles of GA, PIF and KL signaling with germination. To address these comments, we have expanded the Discussion section, added relevant references, and produced a working model clarifying the contributions of KL signaling to germination in the dark versus the light. It is important to note, that this model is limited to *Arabidopsis* germination and may not apply to other developmental stages.
2. In studying the role of *SMAX1* in regulating germination we developed a simplified dark treatment protocol. Our conditions appear to produce seed with inactivated PHYB but reviewers would like this verified by doing experiments using far-red light. We have performed this far-red light experiment and found these conditions gave very similar results to our dark germination results. In summary, we believe our experimental setup presents a simple new protocol for studying germination in the dark for plant researchers. Our red-light experiments are shown in **Figure S2**.

We appreciate the comments and suggestions to improve our manuscript. Please let us know if there are further suggestions and/or edits needed.

Best wishes,

Shelley Lumba Ph.D.

Assistant Professor

University of Toronto

**Reviewer 1**

1. **Why does 20 µM PAC inhibit the germination of *smax1/smxl2* effectively in the dark (Fig 4D), but not *smax1* single mutants in the light (Fig 5)? This group reported that *smax1* mutants can bypass the germination requirement for GA in the light (Bunsick, *Nature Plants*). But these latest data imply that *smax1* cannot do so in the dark. Mechanistically speaking, why? Do *SMAX1/SMXL2* vs GA have a relatively different effect on DELLA levels in light vs dark?**

This comment on differences between light and dark PAC sensitivity is raised by other reviewers. We have substantially expanded the **Discussion** section (**LINES 399-408**) and have included a working model (**Figure 6, see point 3**). Briefly, our evidence suggested that SMAX1 and SMXL2 work through PIF1 to control dark germination. Addition of KL degrades SMAX1 and SMXL2 inactivating PIF1 which in turn increases GA levels and decreases ABA. Increasing GA destabilized DELLA repressors stimulating germination. Hence, in the dark, germination is responsive to PAC addition since depleting seeds of GA ensures that DELLAs remain stable and repress germination. In the light, activated Phytochromes (PHY) inactivates all PIF proteins resulting in inactive DELLA repressors, thus changes in GA levels will have little effect on seed germination. Consequently, in the light, germination is insensitive to PAC addition.

The reviewer also asks about potential differences in the effects of SMAX1 and SMXL2 versus GA on DELLA levels in light versus dark. There are numerous reports of GA and SL levels affecting each other in some form of antagonism. As for a relationship between SMAX1 and SMAX2 with DELLAs, it has been shown that SMAX1 protein directly interacts with RGL1 and RGL3 (Xu et al., Cell Rep 2023). Neither of these proteins impact seed germination extensively but they are related to RGL2, which is a key central repressor of germination in *Arabidopsis*. This interaction appears to enhance SMAX1 transcription and inhibits *GA3ox2* expression, an enzyme involved in GA biosynthesis. Both of these events should decrease germination. Phenotypic analysis of these relationships suggest they could contribute to germination behaviors under low light conditions. We have added this information to the **Discussion** section with **LINES 388-398**.

2. **Perhaps related to the above query, in the Discussion the authors say "Low nanomolar KAR2 concentrations were sufficient to stimulate high germination rates of *AtKAI2OX* seed in the dark, which was unexpected given that KAR2 addition to *AtKAI2OX* seed in the light only partially germinates seeds depleted of GA" (lines 357-358). Perhaps I am missing something obvious, but I don't see the link between the two parts of that sentence - unless the surprising fact (or implication) is that GA is required for germination in the dark, but not the light (as stated in the next sentence)? Maybe rearranging the start of this paragraph, and explaining in a little more depth, would help.**

This is addressed in Point 1.

3. **To this end, I think readers (and myself!) would value a figure that helps to integrate current knowledge of how GA, KAR-KL-SMAX, DELLAs, PIFs and light feed into the decision to germinate. If desired, such a model could take into account the authors' prediction that "SMAX1/SMXL2 is also necessary for PIF1 function in the dark" (line 337).**

We have added a working model in Figure 6, see below.

**Figure 6. A working model on the contributions of SMAX1 and SMXL2 to germination under dark versus light conditions**. Far left panel, in the dark, PIF proteins actively repress light response genes. Under low KL conditions, SMAX1 and SMXL2 activate PIF1 to reduce the GA to ABA ratio discouraging germination. SMAX1 and SMXL2 also could act to directly dampen germination as these proteins interact with EAR motif repressors. Center left panel, increasing KL inhibits SMAX1 and SMXL2 function to both alleviate the repressive effects of these proteins and increase the GA/ABA ratio. Although this allows germination in the dark, inhibition of GA synthesis by addition of the GA synthesis inhibitor, PAC, will decrease the germination response due to the repressive effects of DELLA proteins. Right central panel, in the light, phytochromes inactivate PIF proteins resulting in DELLA protein inactivation. Although seeds no longer require GA to germinate, SMAX1 and SMXL2 proteins still maintain their function to attract EAR repressors. Also, SMAX1 is thought to inhibit PHYB inactivation of PIF4 in the light. This may be true of other PIF proteins. These events would suggest a weaker germination response in low KL conditions. Far right panel. Increased KL levels inhibit SMAX1 and SMXL2 function and this in combination with PIF inactivation of DELLA repressors will lead to a stronger germination response in the presence of PAC.

4. **I was surprised not to find any discussion of Xu et al. (2023) [Cell Reports 10.1016/j.celrep.2023.112740]. This article seems relevant in the context of *SMAX1* signalling via DELLAs. Of note, they did not report any germination of (*max2*) *smax1 smxl2* seeds in darkness (I assume different treatment methodologies), although the triple mutant does germinate more readily under low light and the double is relatively more resistant to PAC.**

We agree with the reviewer and have added this information to the Introduction **LINES 86-89**: with the sentences “*Moreover, SMAX1 protein interacts with gibberellin (GA) repressor proteins RGL1 and RGL3 to inhibit seed germination under weak light conditions [16]. Thus, there is a complex relationship between KL and GA signaling with respect to regulating germination under light-limiting conditions*.”

We have also greatly expanded the Discussion, **LINES 388-408**, to discuss the relationship between GA and SL biosynthesis as well as SMAX1/SMXL2 and DELLAs (outlined in Point 1).

5. **Methods Line 444: "Seeds for dark germination assays were left to dry for at least six months before use." Evidently seed storage protocol is an important aspect of this research. Could the authors please explain in more detail how this drying process was done? The seed were harvested first I assume (or were they left to dry on the plants for six months??), then placed in some sort of container (what sort), at low RH (how)?**

We have added this information to the Methods section. **LINES 504-506** “*Seeds for dark germination assays were left to dry for at least six months in a glass tube and closed cardboard storage box at room temperature (24^0^ C) in the laboratory before use.”*

6. **Methods Line 517, the reference gene was apparently AtACTIN8, but in the legend for Fig 1, it says 18S rRNA. Please check and correct. Neither of these is a particularly good reference gene but the changes in gene expression are considerable and unlikely to be affected meaningfully either way. If they were both used, all the better.**

We have corrected this mistake.

***Minor corrections***

1. **Line 57, Striga is a genus, not a genera**
2. **LINE 59. “Parasitic plants of the *Striga* genus are another example.”**
3. **Line 321 "SMAX1/SMXL2 inhibits" - I know what you mean, and this feels a bit pedantic, but the use of a slash and singular verb implies that SMAX1 and SMXL2 are the same thing - and they aren't quite. How about "SMAX1 and SMXL2 inhibit..." Likewise lines 104, 197 and probably elsewhere as well.**
4. **This was amended. All “SMAX1/SMXL2” was change to “SMAX1 and SMXL2”**
5. **Line 363, seedlings "squelch" PIF proteins? That's a good one. Sequester?**
6. **LINE 407. “Models suggest that increased DELLA protein levels in dark-grown seedlings sequester PIF proteins, which results in skotomorphogenic development”**
7. **Line 503, assembly**

We have made all these corrections.

**Reviewer 2**

1. **Line 135-136: why saying “well characterized lines”? In ref 10 (Bunsick et al, 2020) a single AtKAI2OX line is mentioned (Fig 4); may be cite another reference if both have been described elsewhere? Also, mention which line is shown in Fig1A and D, and used for transcriptomic analysis.**

We previously published these two *AtKAI2OX* overexpression lines in Toh *et al. Science*, 2015 and have added the citation in the Figure 1 legend and within the text. We now specify which overexpression line was used: **LINES 157-159:** “*Consistent with our genetic results, the expression of genes known to be induced by AtKAI2 activation (DLK2, KUF1, BBX20) increased in dark-germinated AtKAI2OX-A seeds treated with KAR_2_ (Fig 1D)*”.

We also modified the Figure 1 legend as follows: **LINES 171-172:** “(D) *Expression (RT–qPCR analysis) of KAR-induced genes (DLK2, KUF1, BBX20) in dark-germinated AtKAI2OX-A seeds on 0.5 µM KAR_2_*.”

2. **Reference to citation (18) (Oh et al, 2004), occurs repeatedly (lines 94, 96, 216, 228, 234, 236, 241…), but not appropriately to my opinion. Unless I missed some figures/tables, and the authors did the transcriptomic analysis of WT vs pif1, this citation (18) is provided as reference for the list of PIF1-regulated genes, for Figure 3 and Figure 4 results (Comparison of Differentially Expressed genes versus AtKAI2OX + KAR2). However, this is confusing, because PIF1 regulated genes are not described in Oh et al, 2004. Therefore, it should be clarified where do pif1 transcriptome data come from***.*

We have corrected this reference where needed to Oh et al., 2009.

3. **In addition to the precise origin of the *pif1* transcriptomic data, it would be useful to indicate the threshold used to distinguish the 61 genes mentioned line 215; this is not obvious to me, even from S3 table*.***

We went back and re-evaluated our transcriptomic data using a cutoff of 2-fold to identify genes responsive to KAR_2_. We then compared this dataset to a gene set containing direct targets of PIF1 as assayed by CHIP seq. We have modified **LINES 232-234** “*Notably, 94 of the 166 known direct targets of PIF1 were activated 2-fold by KAR2 (Fig 3C, S3 Table).”* and have revised Supplementary Table 3.

4. **Line 178-180: for non-specialists and in general for clarity, the use of *rac*GR24 should be explained, and in particular why it is “expected” that the YFP-SMAX1 protein does not accumulate upon the addition of “an artificial SL”. Why not using karrikins? Please indicate the duration of racGR24 treatment. Also, did the authors test the germination of seeds from the *YFP-SMAX1* overexpressing line?**

We have added a description of the issue with GR24 isomers to the **Introduction** section with **LINES 69-76**: “*Presently, the ligand for AtHTL is not identified although this receptor is closely related to other* α/β *hydrolase receptors that bind the hormone strigolactone (SL) [9]. Chemically, canonical SL structures are tricyclic lactones (ABC rings) connected to a butenolide ring (D-ring) via an enol-ether bridge. The stereochemistry around the D-ring is important as only SLs with (+)-(2’R) stereochemistry are bioactive [10]. Surprisingly, unlike SL receptors, AtKAI2 is activated by SLs with (-)-(2’S) stereochemistry [10]. However, because 2’S isomers do not occur in nature, a compound, dubbed KL, for KAI2-ligand, has been hypothesized [9].*

We have also added **LINES 194-198**: “*To assess if the eYFP-SMAX1 line was responding correctly, we reperformed our light shift experiments in the presence of rac-GR24. Since exposure to the (–)-(2’S)-GR24 isomer of rac-GR24 activates AtKAI2 signaling resulting in SMAX1 degradation, the eYFP-SMAX1 signal should decrease in the presence of rac-GR24 independently of light conditions. Indeed, only weak YFP signal was detected in rac-GR24 treated roots under all conditions (****Figure 2A, B****).* As for using *rac*-GR24 versus karrikins this was an economic choice as *rac*-GR24 was more affordable.

Yes, our YFP-SMAX1 transgenic line germinated 100% on MS-agar media.

5. **Line 204-206: Is the reference (24) (Huq et al, 2004) appropriate to support the claim? This reference lays the groundwork for chlorophyll biosynthesis, which is key to the photosynthetic apparatus*.***

Based on the reviewers comments we have modified this sentence to **LINES 220-222**: “*Despite the absence of light stimulus, KAR2-treatment of AtKAI2OX seed upregulated the expression of many genes typically only induced by light (Fig 3A, S2 Table) [Ma L, Li J, Qu L, Hager J, Chen Z, Zhao H, et al. Light Control of Arabidopsis Development Entails Coordinated Regulation of Genome Expression and Cellular Pathways. Plant Cell.2001].*”

6. **Figure 5, and line 285 clustering of candidate mutants: The rationale for testing PAC + *rac*-GR24 should be clarified. Why not using KAR2?**

Because of this comment and Point 7, we have rewritten this genetic screening section of the results for better clarity. Again, we did not use KAR_2_ for these experiments mostly for economic reasons as these screens required large amount of chemical. With this said, we wanted to know if mutants insensitive to PAC would show the same response in the presence of *rac*-GR24. To this end, we found a large block of PAC resistant mutant lines, including *smax1-2,* showed less resistance to PAC in the presence of *rac*-GR24.

Although we do not understand the nature of this phenotype, it may reflect genetic redundancy through SMXL2 and other pathways such as D14-dependent signaling. We have clarified the rationale of PAC + *rac*-GR24 with the following **LINES 306-308:***“We also included a condition of PAC plus rac-GR24 to see if any PAC insensitive mutants could be rescued by addition of this artificial SL/KL.”*

7. **Line 300, please clarify the sentence, or provide another citation: in ref (32) Cotylimide-VI is described as perturbing SL accumulation, but no mention is made of ABA/GA ratios. Why would “SL constitutive mutants” be found if the screen is made on seed germination (KAI2 pathway)? To note, the Bleaching/CTL-VI sensitivity of WT is not indicated on Fig 5 (mentioned line 302)**

CTL-VI bleaches wild type cotyledons and SL mutants are less sensitive to this bleaching effect. It was originally interpreted that plants with decreased SL synthesis or signalling are resistant to CTL-VI. In retrospect, the sensitizing effect of CTL-VI is most likely through increased KL signaling and the effects we see are due to the (–)-(2’S)-GR24 isomer. We predict that *smax1* loss-of-function mutants should be hypersensitive to *rac*-GR24 as they are constitutive for KL signalling, which was the phenotype we observed.

We have rewritten this section for clarity **LINES 324-330**: “*Mutants perturbed in KL signaling can be identified using a compound called cotylimide-VI (CTL-VI) [36]. Addition of CTL-VI to germinating Arabidopsis seed bleaches their cotyledons and max2 mutants, which are insensitive to SL or KL signaling are insensitive to this bleaching effect [36]. We, therefore, thought mutants constitutive for KL signaling, like smax1-2, may show increased sensitivity to CTL-VI application versus ABA auxotrophs with respect to cotyledon bleaching. Indeed, CTL-VI preferentially bleaches smax1-2 cotyledons exposed to rac-GR24 compared to aba2-2 and abi4-5 cotyledons.”*

Finally, we have also indicated WT sensitivity on the figure.

8. **Line 353: which ligand is it about? *rac*-GR24 has been used but what is known about SL or KL/KAR sensitivity to light**?

Because KL has not been identified and we are not sure if KARs are metabolized in the dark vs light, we decided to not speculate on the role of ligands in AtKAI2 sensitivity. To clarify, we have expanded this section in **LINES 379-385**: “*Unfortunately, the absence of a KL ligand candidate limits speculation on how light conditions may affect this ligand composition, concentration or sensitivity. Whatever the case, special consideration regarding the development stage of the plant and light regime should be given when studying SMAX1 stability*.

9. **Line 368-369: it is not very clear to me what the authors want to say here.**

We have removed this line.

10. **The Abstract last sentence is very attractive but does not really reflect the work presented.**

We have amended this in **LINES 37-39**. “*The interaction of HTL/KAI2 and light signalling may explain how specialist plants like ephemeral and parasitic weeds evolved their germination behaviour in response to specific environments*.”

**Minor Comments:**

**Line 250-251, please clarify the sentence (“and consequently SOM”?).**

*SOM* refers to the *SOMNUS* gene, which we defined earlier in the paragraph. We have clarified this section to **LINES: 265-268**: *“Unlike SOM, however, AtKAI2 activation induced expression of a different CYP707 homolog (CYP707A3 versus CYP707A2) and decreased expression of GA2ox2 (Fig 4B). Even considering this nuance, the transcript profile of KAR_2_ activation of AtKAI2, demonstrated an overall similarity to the transcript profile of a pif1l loss-of-function dark-treated seeds****.****”*

**Line 330: remove “by”**

We have removed “by”.

**Line 372: about the fact that light likely stimulates AtKAI2 expression: is it possible to find data that confirm this? in Waters et al Plant Cell 2012, KAI2 transcript levels in seedlings are strongly induced by light.**

We have added **LINES 379-385** and references (Waters et al., 2012) to this section:

“*Unfortunately, the absence of a KL ligand candidate limits speculation on how light conditions may affect this ligand composition, concentration or sensitivity. However, light and the addition of KARs or rac-GR24 stimulate AtKAI2 transcription, which is consistent with our findings that SMAX1 is degraded in the light during early stages of germination [Waters et al., 2012; 12,13]. Whatever the case, special consideration regarding the development stage of the plant and light regime should be given when studying SMAX1 stability.”*

**Lines 441 and 460: probably refer to another work? Please correct.**

The reference on Line 441 [6] on the original submission is correct. There is no reference on the original submission on line 460.

**Line 143: please cite a reference for these genes “known to be induced by AtKAI2 activation”.**

We have added citations **LINES 157-159**: “*Consistent with our genetic results, the expression of genes (DLK2, KUF1, and BBX20) known to be induced by AtKAI2 activation increased in dark-germinated AtKAI2OX-A seeds treated with KAR_2_ (Fig 1D****)*** *[Stanga et al., 2016; Wei et al., 2016: 24, 25].”*

**Line 156 (legend to figure 1) and methods: How many seeds per replicate? I could not find the information in tables nor in material and methods.**

This was added to the Methods section **LINE 529-531** “*Approximately 25 to 35 of unsterilized dry seeds per replicate were sprinkled directly onto 10 mm petri plates in the dark containing water agar (0.8%) and the fungicide benomyl (10 µg/ml) (Toronto Research Chemicals, B161380).*”

**Line 158 (legend to figure 1): the method section mentions the use of ACTIN 8 (line 517), not 18S rRNA, please correct.**

This was amended and discussed as suggested by Reviewer 1 Point 6.

**Line 238: why is SOM qualified as “novel”?**

We used “novel” to describe a protein that has no obvious function based on gene identity. We have removed it.

**Reviewer 3**

1) **The manuscript should try to better integrate prior research on KAR signaling, light, and GA during germination. Some of the findings here (as a couple examples, regulation of GA3 oxidases and light-regulated genes in darkness, enhancement of germination by KARs under highly light-limited conditions) by KAR signaling overlap with those made in prior studies but this is not acknowledged or discussed with any meaningful depth.**

This comment on differences between light and dark PAC sensitivity is raised by other reviewers. We have substantially expanded the **Discussion** section (**LINES 399-408**) and have included a working model (**Figure 6**). Briefly, our evidence suggested that SMAX1 and SMXL2 work through PIF1 to control dark germination. Addition of KL degrades SMAX1 and SMXL2 inactivating PIF1 which in turn increases GA levels and decreases ABA. Increasing GA destabilized DELLA repressors stimulating germination. Hence, in the dark, germination is responsive to PAC addition since depleting seeds of GA ensures that DELLAs remain stable and repress germination. In the light, activated Phytochromes (PHY) inactivates all PIF proteins resulting in inactive DELLA repressors, thus changes in GA levels will have little effect on seed germination. Consequently, in the light, germination is insensitive to PAC addition.

The reviewer also asks about potential differences in the effects of SMAX1 and SMXL2 versus GA on DELLA levels in light versus dark. There are numerous reports of GA and SL levels affecting each other in some form of antagonism. As for a relationship between SMAX1 and SMAX2 with DELLAs, it has been shown that SMAX1 protein directly interacts with RGL1 and RGL3 (Xu et al., Cell Rep 2023). Neither of these proteins impact seed germination extensively but they are related to RGL2, which is a key central repressor of germination in *Arabidopsis*. This interaction appears to enhance SMAX1 transcription and inhibits *GA3ox2* expression, an enzyme involved in GA biosynthesis. Both of these events should decrease germination. Phenotypic analysis of these relationships suggest they could contribute to germination behaviors under low light conditions. We have added this information to the **Discussion** section with **LINES 388-398**.

**Figure 6. A working model on the contributions of SMAX1 and SMXL2 to germination under dark versus light conditions**. Far left panel, in the dark, PIF proteins actively repress light response genes. Under low KL conditions, SMAX1 and SMXL2 activate PIF1 to reduce the GA to ABA ratio discouraging germination. SMAX1 and SMXL2 also could act directly to dampen germination as these proteins interact with EAR motif repressors. Center left panel, increasing KL inhibits SMAX1 and SMXL2 function to both alleviate the repressive effects of these proteins and increase the GA/ABA ratio. Although this allows germination in the dark, inhibition of GA synthesis by addition of the GA synthesis inhibitor, PAC, will decrease the germination response. Right central panel, in the light, phytochromes inactivate PIF proteins resulting **in** DELLA protein inactivation. Although seeds no longer require GA to germinate SMAX1 and SMXL2 proteins still maintain their function to attract EAR repressors. Also, SMAX1 is thought to inhibit PHYB inactivation of PIF4 in the light. This may be true of other PIF proteins. These events would suggest a weaker germination response in low KL conditions. Far right panel. Increased KL levels inhibit SMAX1 and SMXL2 function and this in combination with PIF inactivation of DELLA repressors will lead to a stronger germination response in the presence of PAC.

2) **Some species have positive germination responses to light; others are negatively photoblastic. This is a separate feature from KAR-responsiveness, as some negatively photoblastic species are stimulated by KARs. This should be considered in the discussion and elsewhere (for example, line 43 implies that all "generalist" plants respond positively to light).**

While it is important to indicate that not all species are positively photoblastic the majority of plants do use light as a positive germination signal and for this reason, we believe light-dependent germination is the more generalist lifestyle. It this context, it could be argued that negatively photoblastic germination is a specialist response. Of course, negatively photoblastic species are interesting, but we believe adding this complexity is beyond the scope for this manuscript.

3) **The authors should perform a germination experiment with far-red light treatment of Col-0 and *smax1 smxl2* to determine whether they have achieved true darkness. Although the light during plating was dim (line 467), there was still light. In addition, phytochrome could have been activated in dry seed during storage or between storage and plating; while this may not have had much effect on wildtype seed, the *smax1 smxl2* seed are predisposed to germination and have a lesser light requirement. Without doing this experiment, statements such as "activating the *AtKAI2* pathway in the dark completely bypasses the requirement of light for germination in Arabidopsis" (line 79-80) will need to be less bold.**

We agree and tested germination under red-light conditions. The results were very similar to our previous light conditions indicating our simplified germination experimental set-up is useful to researchers (**S2 Fig, see below**). We have added **LINES 128-130: “*We tested this by giving seeds a five-minute pulse of far-red light and found similar germination*** *responses to our dark conditions (S2 Fig)*.”

In addition, we have removed the phrase, **"**activating the *AtKAI2* pathway in the dark completely bypasses the requirement of light for germination in Arabidopsis" from the manuscript.

***S2 Fig. Activation of the AtKAI2 signalling pathway can germinate seeds in PhyB-off conditions.*** *(A) Representative images of seeds treated with or without 0.5 μM KAR2 after five minutes of far-red light exposure and placed in a room-temperature dark cabinet. (B) Germination percentages of seeds after a far-red light regime as shown in (A). Germination was counted as radicle emergence. Bar plot represents three biological replicates. Black circles represent the mean of each biological replicate. Bars represent SD.*

4) **In construction of the double mutant *htl-3 pif1-1* (line 460), how was it verified that *ShHTL7OX* transgene was absent?**

Our *ShHTL7* transgenes are tightly linked to the BASTA resistance transgene, so we verified lines lacking *ShHTL7* by ensuring they were 100% sensitive to BASTA. We have modified our Methods section as follows: **LINE 539-546**: “Putative *htl-3; pif1* double mutants were identified from F_2_ offspring of *htl-3; ShHTL7OX* x *pif1* cross by first screening for the *pif1* dark germination phenotype. Plants were then screened for the *htl-3* round rosette leaves and long hypocotyl phenotype. These lines were also screened on glufosinate ammonium (BASTA) to look for lines that were 100% sensitive on BASTA to ensure that the *ShHTL7OX* transgene was absent. Potential *htl-3; pif1* homozygous lines were confirmed by genotyping of individual plants by PCR for the *htl-3* and *pif1* allele. Primers used for genotyping are listed in S6 Table. See S5 Fig for workflow and PCR gels confirming *htl-3; pif1* double mutants.”

Additionally, we have amended our manuscript to accurately describe which *pif1* allele (*pil5-1*) was used for genetic crossing. Attached here are images of PCR gels confirming homozygosity of the *htl-3; pif1* double mutant with allele-specific primers. We have added a Supplementary Figure (S5 Fig) in our methodology of the workflow used to confirm homozygous *htl-3; pif1* double mutants with PCR.

5) **The *smax1-2; 35S::eYFP-SMAX1* line was analyzed by confocal microscopy in the T2 generation, which is segregating for the transgene. Was this a single-insertion transgene? Was PAC and hygromycin treatment used prior to imaging (line 523)? If so, is it certain that these treatments do not have an effect on SMAX1 stability? Were negative controls (non-transgenic segregants in the same pool) imaged as well? How were they distinguished?**

The T2 generation was tested on PAC and hygromycin treatment prior to imaging. This was to ensure our line, which complemented our genetic background (*smax1-2*), conferred resistance to hygromycin. The segregation ratio of the transgenic line was 15:1, indicating this line contains a double insertion of the transgene (see S3A Fig). Hence, this double insertion should have at least one copy of the transgene in 15 out of 16 seedlings we imaged. Additionally, we imaged our negative control, *smax1-2* with no transgene and never detected YFP signal. Therefore, we concluded nuclear localized YFP signal is a true signal and seedlings accumulating this signal are expressing our transgene.

Before imaging, the seedlings were not treated with any chemicals to ensure the stability of SMAX1 in the dark was not confounded by any treatment (PAC and hygromycin). Our conditions for germinating our transgenic line is outlined in our Methods. The *rac-*GR24 treatment was applied to the seeds throughout stratification, germination and light or dark treatment. Therefore, we believe that it is appropriate to image the T2 generation of our transgenic line.

6) **For the mutant screen (line 530), provide details on the EMS treatment (e.g. concentration and duration) and approximate number of M1 plants, which will have implications for the degree of saturation of the screen.**

This following statement has been added to the Methods section. **LINES 587-588**: “*To generate a M_1_ EMS population, approximately 20,000 seeds were mutagenized in 0.2% for 16 hours and then washed in distilled water 10 times before sowing onto soil*.”

7) **line 68 - clarify "natural SL enantiomers**"

We have addressed this issue in Reviewer 2 Point 4. As mentioned, we have added to the **Introduction**: **LINES 69-76** *“Presently, the ligand for AtHTL is not identified although this receptor is closely related to other a/b hydrolase receptors that bind the hormone strigolactone (SL) [7, 23]. Chemically, canonical SL structures are tricyclic lactones (ABC rings) connected to a butenolide ring (D-ring) via an enol-ether bridge. The stereochemistry around the D-ring is important as only SLs with (+)-(2’R) stereochemistry are bioactive [23]. Surprisingly, unlike SL receptors, AtKAI2 is activated by SLs with (-)-(2’S) stereochemistry [23]. However, because 2’S isomers do not occur in nature, a compound, dubbed KL, for KAI2-ligand, has been hypothesized [8].*

8) **line 71 - what qualifies as a "minor role" in germination?**

We expanded on this sentence and added references. **LINES 77-79**: “*Although loss-of-function mutations in AtKAI2 result in increased primary dormancy in some Arabidopsis accessions, AtKAI2 at first glance, appears to play a minor role in germination of after-ripened seed* [Nelson et al. 2009, Waters et al., 2012 [11, 12, 13].”

9) **line 93-94 - how does decreasing the level of ABA promote dormancy? this seems backwards**

We have corrected this sentence as follows, **LINES 103-106**: *“One of the functions of PIF1 is to modulate the levels of a variety of hormone signalling and response genes. Inactivation of PIF1 decreases the level of abscisic acid (ABA), which would decrease seed dormancy, and increases the level of GA, which should promote germination [21]”*.

10) **line 117 - is soil devoid of nutrients?**

We have now modified this sentence as follows, **LINES 130-132**: “*Second, seeds were plated on water and agar media devoid of nutrients and a carbon source, which removes the promotive effects of nitrogen and carbon on germination.*”

11) ***Show evidence in the supplement that benomyl has no effect on germination or seedling viability (line 126-127).***

The choice of benomyl was based on commonly performed protocols for floral transformation of *Arabidopsis*. We have, however, added a supplementary Figure (**S1B Fig**) to show that plating on water-agar media with or without benomyl did not affect germination in the dark.

12) **line 147 - this does not logically follow line 146; there may be other ways to stimulate germination in the light than SMAX1/SMXL2 inactivation even if SMAX1/SMXL2 repress germination in the dark**

We have modified the sentence to **LINES 160-161**: “*Our results indicated that SMAX1 and SMXL2 contributes to the repression of germination of Arabidopsis seeds in the dark.”*

13) **Figure 1B - Is the minimal KAR2 concentration on the x-axis 0.1 nM or is it supposed to be 0? Either way, the data points for this minimal concentration are missing. (Presumably they exist based on the curve fitting)**

We have adjusted our x axis and graph on Fig. 1A to reflect our data points more clearly.

14) **Various figures - It is not appropriate to use boxplots for small sample sizes (e.g. n=3). There is insufficient data to determine the quartiles for a boxplot. Instead, show the individual data points and the summary statistic (e.g. mean, optionally with SD or SEM bars). For Figure 1D, it is also inappropriate to claim a sample size of 9 (Table S1). From the methods, it sounds like there were three biological samples that were assayed by qPCR with three technical replicates. Use the average of the three technical replicates for each biological sample and show the data for the n=3 biological samples.**

We thank the reviewer for bringing this to our attention and have adjusted the graphs to better represent the datapoints. We have also updated our supplementary tables with our summary statistics.

15) **line 180 - *rac*-GR24 is more than just an artificial SL; its component stereoisomers have different activities**

This is addressed in Reviewer 2 point 4 with the sentence, **LINES 202-206**: “*To assess if the eYFP-SMAX1 line was responding correctly, we reperformed our light shift experiments in the presence of rac-GR24. Exposure to rac-GR24 activates AtKAI2 signaling through the (-)-(2’S) isomer and should result in SMAX1 degradation. Indeed, only weak YFP signal was detected in rac-GR24 treated roots under all conditions (Figure 2A, B).*

16) **line 233 - "nexus for dialling up the combination of hormonal responses" is unclear; please rephrase**

We have modified the sentence to **LINES 259-261**: “*Since many of the genes downstream of PIF1 are annotated as responsive to hormones, PIF1 appears to contribute to establishing the correct combination of hormonal responses required for germination [18]*”.

17) **PAC application blocked germination of smax1 smxl2 (line 263); discuss why this result is different from Bunsick et al., 2020, Nature Plants "SMAX1-dependent seed germination bypasses GA signalling in Arabidopsis and Striga"**

We have greatly expanded our explanation of this response and generated a working model, Figure 6. Please refer to Point 1.

18) **line 323 - it is inaccurate to say that the role of AtKAI2 signaling in germination is "only uncovered when seeds are artificially depleted for GA"; there are many studies which have shown the role of this pathway in germination without depleting GA**

We have removed these sentences*.*

19) **line 328-329 - this is confusing; consider rephrasing**

We have rephrased this to **LINES 368-373**, “*By contrast, fire-following ephemeral weeds and some parasitic plant species have an absolute requirement for HTL/KAI2 for germination and are not responsive to GA [3,7,8,11].* ***We believe we have reconciled these species differences by showing that Arabidopsis germination like parasitic and ephemeral weeds can also become completely dependent on AtKAI2 signaling under appropriate environmental conditions****.”*

20) **line 372 - while it is of course impossible to say at this time how light affect KL production, I suspect there is published data on how light affects AtKAI2 transcript or protein abundance**

We agree KL production is likely light-dependent but as there is no evidence for this since the identify of KL is unknown, it is probably premature to dwell on this subject. Instead, we have added **LINES** **395-401**. “*Unfortunately, the absence of a KL ligand candidate limits speculation on how light conditions may affect this ligand composition, concentration or sensitivity. However, light and the addition of KARs or rac-GR24 stimulate AtKAI2 transcription, which is consistent with our findings that SMAX1 is degraded in the light during early stages of germination [12, Waters et al., Development 2012]. Whatever the case, special consideration regarding the development stage of the plant and light regime should be given when studying SMAX1 stability.”*

21) **line 421-425 - I disagree with the validity of these statements and this analysis.**

Since the reviewer did not specify why and with which statements and analysis they disagreed, it is very difficult for us to address this comment.
