# Supplementary material for: HTL/KAI2 signaling substitutes for light to control plant germination
Source: PLoS Genet. 2024 Oct 21;20(10):e1011447. doi: 10.1371/journal.pgen.1011447 (PMC11527322; doi:10.1371/journal.pgen.1011447)
Supplement: Attachment — Submitted filename: renamed_28a95.docx [file pgen.1011447.s015.docx]

This manuscript details the important role of SMAX1 in Arabidopsis germination in the dark and provides evidence that both SMAX1 and SMXL2 are necessary for PIF1 function under these conditions. A working model is presented to describe the contributions of SMAX1 and SMXL2 to germination in dark versus light conditions, effectively integrating prior research on KAR signaling, light, and GA during germination. These results are significant and impactful in explaining how specialized plants, such as ephemeral and parasitic weeds, have evolved their germination behavior in response to specific environments.

Major comments：

1. Fig 5, the photographs showing the bleached seedling phenotype of various mutants grown on 2.5 µM CT-VI are not clear enough, possibly due to insufficient image clarity or small size. So I don't think it is obvious that "mutants constitutive for KL signaling, like smax1-2, may show increased sensitivity to CTL-VI application compared to ABA auxotrophs with respect to cotyledon bleaching." Please present the results of CTL-VI treatment on the mutants in a separate figure.
2. Why was the expression of genes known to be induced by AtKAI2 activation (DLK2, KUF1, BBX20) lower in dark-germinated AtKAI2OX-A seeds treated with DMSO than in wild-type seeds (Fig 1D)?
3. Could the author please explain how to treat *smax1-2; smxl2-1* seed with Abscisic acid (ABA) and paclobutrazol (PAC)（S4 Fig）？
4. Please explain why smax1-4 germinated well in the dark while smax1-2 did not germinate well in the dark (Fig 5).
5. Line 423-424 (Fig 6) in the Revised Article with Changes Highlighted version: Which EAR repressors are recruited by SMAX1 and SMXL2 proteins? Please provide suitable references.
6. Why are smax1-2; 35S::eYFP-SMAX1 seeds sprinkled onto ½ MS (Murashige-Skoog) agar (0.8%), while the other seeds in the Dark Germination Assays are sprinkled directly onto Petri plates in the dark containing water agar (0.8%) and the fungicide benomyl (10 µg/ml)?

Minor comments：

1. **Line 188 of the Revised Article with Changes Highlighted version:** The reference to "S2 Fig" is incorrect. "S2 Fig" does not mention PAC treatment.
2. Line 277：S4 Fig，not S3 Fig.
3. Line 327: "Mutants perturbed in KL signaling can be identified using a compound called cotylimide-VI (CTL-VI) [36].” Should this reference be to citation 37 instead of 36? Please confirm.
4. Line 502：“*Col-0*” should not be written in italics.
5. Line 503: “smax1-2/smxl2-1 [38] pif1-1 [21]”, a comma is missing here.
6. Line 504:“abi4-5 [53], cop1-4 [53], aba2-2 [54], abi1-1 [51], det1-1 [55]”All genotypes in this sentence should be italicized.
7. Line 506: Please clarify the sentence, “All seeds were grown in a continuous 24-hour light chamber at 22°C.” What kind of photoperiod does Arabidopsis thaliana require for growth?
8. Line 509:“**24^0^ C**”The unit of Celsius is misspelled.
9. Line 507-509：“Seeds for dark germination assays were left to dry for at least six months in a glass tube and closed cardboard storage box at room temperature (**24^0^ C**) in the laboratory before use.”This sentence should be moved to the method part of “Dark Germination Assays”.
10. Line 535-537:“Approximately 25 to 35 of unsterilized dry seeds per replicate were sprinkled directly onto 10 mm petri plates in the dark containing water agar (0.8%) and the fungicide benomyl (10 µg/ml) (Toronto Research Chemicals, B161380). Unsterilized dry seeds were sprinkled directly onto 10 mm petri plates in the dark containing water agar (0.8%) and the fungicide benomyl (10 µg/ml) (Toronto Research Chemicals, B161380).” This passage contains a repeated sentence.
